# A Rare Case of Primary Amenorrhea with Two Etiologies, Hypothalamic Amenorrhea, Transverse Vaginal Septum, and No Hematocolpos

**DOI:** 10.1155/2015/989123

**Published:** 2015-02-16

**Authors:** Firouzeh Ghaffari, Fatemeh Keikha, Arezoo Arabipoor

**Affiliations:** ^1^Department of Endocrinology and Female Infertility at Reproductive Biomedicine Research Center, Royan Institute for Reproductive Biomedicine, ACECR, Tehran 1665659711, Iran; ^2^Department of Obstetrics and Gynecology, Faculty of Medicine, Tehran University of Medical Sciences, Tehran, Iran; ^3^Vali-e-Asr Reproductive Health Research Center, Tehran University of Medical Sciences, Tehran, Iran

## Abstract

We reported a rare case of hypothalamic amenorrhea and transverse vaginal septum. A 28-year-old woman presented with primary amenorrhea and no complaint of abdominal pain. Laparoscopy revealed a small rudimentary uterus with streak ovaries and a vaginal pouch. The patient with diagnosis of Mayer-Rokitansky-Kuster-Hauser (MRKH) syndrome was subjected to a vaginoplasty in another fertility center. In our institute, after two courses of estrogen and progesterone, sonography revealed hematocolpos, while, under anesthesia, transverse vaginal septum was resected. Hysteroscopy revealed normal uterine cavity. She became pregnant 5 months postoperatively with controlled ovarian stimulation (COS) in conjunction with intrauterine insemination, and she has two healthy babies now. This case highlights the importance of careful evaluation of all primary amenorrheas. Clinicians should be aware of presence of more than one etiology which causes atypical presentations and accomplishes a systematic strategy for the evaluation of amenorrhea potential to avoid long-term side effects of a misdiagnosis.

## 1. Introduction

The recommended evaluation for amenorrhea aimed to divide the reproductive system into its components—the genital outflow tract and uterus, the ovary, the pituitary, and the hypothalamus—and to assess the functional integrity of each, starting at the lowest level (the genital outflow tract) and progressing to the higher levels (hypothalamus) of the system until the cause is found [1]. The most common etiologies of primary amenorrhea are the presence of gonadal dysgenesis and hypothalamic amenorrhea (40% and 30%, resp.) [2]. The genital examination is abnormal in 15% of patients with primary amenorrhea [2]. In rare condition, more than one component of hypothalamus-pituitary-ovary (HPO) axis and genital tract are affected. We reported a case of primary amenorrhea with normal secondary sex by Tanner staging, a transverse vaginal septum, and hypothalamic amenorrhea.

## 2. Case

**Figure 1:**
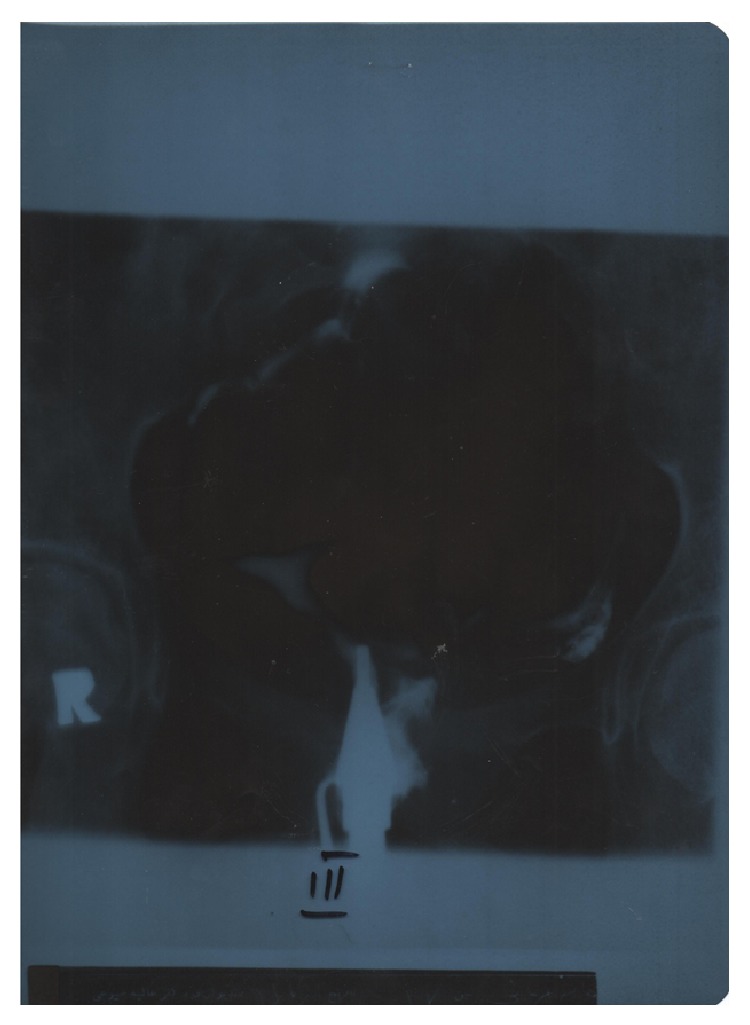
Hysterosalpingography demonstrates rudimentary uterus with streak ovaries, a vaginal pouch, and the absence of cervix.

A 28-year-old nulligravid female presented to our infertility clinic with an 8-year history of primary infertility, sexual dysfunction, and primary amenorrhea. Her body mass index (BMI) was 26 Kg/m^2^. The woman exhibited normal secondary sexual characteristics, normal auxiliary and pubic hairs, normal breast development, and agenesis of the upper two-thirds of the vagina, but there was a vaginal pouch without obvious bulging of the hymen. There was no evidence for stress or changes in weight. She was not a professional athlete and never had abnormal eating habits or complains of abdominal pain. Laparoscopy was performed 6 years ago revealing a rudimentary uterus with streak ovaries and a vaginal pouch, whereas the cervix did not exist (Figure 1). The patient was subjected to a vaginoplasty involving dissection of a space in the upper part of the vagina, which was then subsequently covered by Interceed [3] four years ago. At clinical evaluation, her laboratory testing showed follicle stimulating hormone (FSH) level of 0.2 mIU/mL and luteinizing hormone (LH) level of 0.3 mIU/mL. Furthermore prolactin level, thyroid function tests, and serum androgen level were also within normal limits and her karyotype was 46, XX. The patient was advised to receive oral conjugated estrogens (Aboureihan, Iran) 1.25 mg/day in addition to medroxyprogesterone acetate (5 mg bid; Aboureihan, Iran) 10 mg/day for the last 10 days of one-month estrogen therapy to prevent osteoporosis. The patient was subjected to surrogacy. After two courses, three-dimensional ultrasound confirmed a 56 mm × 27 mm × 42 mm midline uterus with an endometrial lining of 7 mm, while right ovary was 27 mm × 19 mm and left side was 22 mm × 18 mm. There was a cystic area between transverse vaginal septum and external cervical os with ground-glass appearance that indicated old blood in upper vagina. The transverse septum thickness was measured 1.5 mm. Exam under anesthesia and needle aspiration of the vaginal vault revealed “old blood” (hematocolpos). Afterward transverse vaginal septum was resected and, at the same time, hysteroscopy was performed to reveal normal cervical canal and normal uterine cavity. Two hours after surgery, the patient was released without any problem. Eight weeks postoperatively, a Pederson speculum could be admitted. The patient postoperatively received hormone replacement therapy. Four weeks later, sexual intercourse was referred to by both partners as satisfactory. Infertility workup for the husband showed abnormal sperm analysis including a count of 13 million/mL, 50% progressive motility, and 3% normal morphology according to World Health Organization (WHO) criteria. The patient was elected to undergo a controlled ovarian stimulation (COS) in conjunction with intrauterine insemination (IUI) 4 months postoperatively. Tubal patency was confirmed by hysterosalpingography. Human menopausal gonadotropin (hMG; Menogon, Ferring, Copenhagen, Denmark) was used for follicular stimulation. We assessed endometrial thickness and follicular development by transvaginal ultrasound. After 7 stimulation days (21 ampules, 75 IU per ampule), ultrasound showed right ovary with four follicles of 18 mm, 15 mm, 14 mm, and 13 mm, respectively, and left ovary with one follicle of 11 mm. Intramuscular injection (IM) of 5,000 IU human chorionic gonadotropin (hCG; Pregnyl; Darou Pakhsh Pharmaceutical, Tehran, Iran) was given as the final ovulation maturation. A single IUI was planned 36 hours later. The luteal phase was supported with 400 mg/day vaginal progesterone (Abureihan Pharmaceutical Co., Tehran, Iran). Serum beta-hCG (*β*-hCG) concentration was 289 IU/mL on day 12 after insemination, and twin intrauterine pregnancy with fetal heart beat was observed by transvaginal ultrasonography in sixth week of gestation. The patient was followed up by telephone and had cesarean delivery at 36th week of gestation. Now, she has two healthy babies.

## 3. Discussion

Typically, distal occlusions of female genital tract are associated with hematocolpos or hematometra which are manifested with cyclic pelvic pain and amenorrhea [4]. This is considered as a rare case due to presence of a distal obstruction, without abdominal pain and hematocolpos. As reported by Homa et al., the location of septum can influence the timing and type of presentation [4]. The location of septum in present case was in the upper two-thirds of the vagina, which occurs in less than 15 percent of patients [4]. Our patient was misdiagnosed with MRKH syndrome in another fertility center because uterine growth had been stopped due to estrogen deficiency caused by hypogonadotropic hypogonadism (HH), leading to lack of cyclic pelvic pain due to amenorrhea and symmetrical age-appropriate secondary sexual development that was coincident with blind vagina. If low level of plasma gonadotropin, small uterus referred to as hypogonadal state, and estrogen and progesterone withdrawal bleeding were considered earlier, she would not be misdiagnosed with MRKH syndrome and would not suffer from hypogonadism side effects, infertility, and sexual dysfunction.

Evaluation of primary amenorrhea begins with a careful history and physical examination including the assessment of the internal and external genitalia as well as determination of FSH, thyroid stimulating hormone (TSH), and prolactin concentrations. This approach will identify the most common causes of amenorrhea [2]. Primary amenorrhea with a blind or absent vagina points directly to an anomaly of the genital outflow tract [1]. Androgen insensitivity syndrome (AIS), Müllerian agenesis or MRKH, or transverse vaginal septum is considered in patient with appropriate breast development [5], which can be easily distinguished from each other with careful history and physical examination and few laboratory tests. MRKH is seen in approximately 10% of cases of primary amenorrhea [2]. MRKH resulted from Müllerian agenesis or hypoplasia that is followed by absence of the vagina and associated variable uterine development [5]. The uterus and cervix are often absent; however, 7–10% of patients have a rudimentary uterus with functional endometrium [6]. Patients with MRKH are seen with primary amenorrhea as their only complaint, whereas breast and pubic hair development is normal [7].

Patients with AIS have a normal male karyotype (46,XY) and asymmetrical secondary sexual development (breast development with absent pubic hair), no visible cervix, and a short vagina [1]. Transverse vaginal septum and imperforate hymen have been reported in 3%–5% of patients with amenorrhea [2]. Patients present with complaints of cyclic abdominal or pelvic pain because of obstructed menses at the age of menarche. Transverse vaginal septum most often is between the middle and upper third of the vagina [8] but can occur anywhere in the vaginal canal. In septum, a small or a large part of the vagina may be involved [9].

HH is manifested by primary or secondary amenorrhea and normal or low serum gonadotropins concentration [1]. HH is a rare cause of infertility only affecting a small number of patients [10]. This disorder usually is followed by rigorous physical, nutritional, or emotional stress, while breast development ranges from no breast development to moderate breast development [2]. Regardless of the etiology, the patients with HH suffer from amenorrhea, osteopenia, and infertility.

In conclusion, clinicians should be aware of presence of more than one etiology which causes atypical presentations and accomplishes a systematic strategy for the evaluation of amenorrhea potential to avoid long-term side effects of a misdiagnosis.
